# Laterally Movable Triple Electrodes Actuator toward Low Voltage and Fast Response RF-MEMS Switches

**DOI:** 10.3390/s19040864

**Published:** 2019-02-19

**Authors:** Yasuyuki Naito, Kunihiko Nakamura, Keisuke Uenishi

**Affiliations:** 1 Institute for Energy and Material Food Resources, Technology Innovation Division, Panasonic Corporation, Kyoto 619-0237, Japan; nakamura.kunihiko@jp.panasonic.com; 2 Department of Management of Industry and Technology, Graduate of School of Engineering, Osaka University, Osaka 561-0871, Japan; uenishi@mit.eng.osaka-u.ac.jp

**Keywords:** RF-MEMS, switch, capacitor, laterally movable triple electrodes, low voltage, fast response

## Abstract

A novel actuator toward a low voltage actuation and fast response in RF-MEMS (radio frequency micro-electro-mechanical systems) switches is reported in this paper. The switch is comprised of laterally movable triple electrodes, which are bistable by electrostatic forces applied for not only the on-state, but also the off-state. The bistable triple electrodes enable the implementation of capacitive series and shunt type switches on a single switch, which leads to high isolation in spite of the small gap between the electrodes on the series switch. These features of the actuator are effective for a low voltage and fast response actuation in both the on- and off-state. The structure was designed in RF from a mechanical point of view. The laterally movable electrodes were achieved using a simple, low-cost two-mask process with 2.0 µm thick sputtered aluminum. The characteristics of switching response time and actuation voltage were 5.0 µs and 9.0 V, respectively.

## 1. Introduction

Radio frequency micro-electro-mechanical systems (RF-MEMS) switches have a high potential to evolve terminals for wireless communication and wireless sensor networks. Superior performances in RF front-end involve low insertion loss, high isolation, and linear characteristics compared with conventional GaAs FET (field effect transistor) and PIN (P-intrinsic-N) diode switches. The low insertion loss contributes to high receiving sensitivity and low power consumption for mobile terminals. MEMS switched capacitors are also applied to reconfigurable circuits that enable mobile terminals to adapt to multi-band systems [1,2,3]. 

Actuation forces used in the RF-MEMS switches are representatively electrostatic, piezoelectric, electromagnetic, and thermoelastic. The electrostatic actuator is the leading candidate because of its simple structure, CMOS (complementary metal-oxide-semiconductor) compatible process, and high generated force. In spite of these advantages, the RF-MEMS switches using the electrostatic actuator have disadvantages of relatively slow switching speeds over 10 µs, high actuation voltages over 10 V, mechanical reliability, and larger footprint size than the semiconductor switches. They obstruct the RF-MEMS switches to be used in the wireless communication terminals. Low actuation voltage makes switching speed slow, on the other hand, a fast switching speed needs high actuation voltage [4,5,6,7]. The past reported works of lower actuation voltage than 10 V, and a laterally movable actuator, the response time is still over 10 µs [8,9,10,11,12]. The other type of rotary actuator was also reported [13]. The trade-off relation between the switching speed and the actuation voltage makes it difficult to solve undesirable actuation characteristics.

For low voltage actuation and fast response in RF-MEMS switches, it is effective to reduce the spring constant of membrane used for the actuator. However, lower mechanical restoring force due to the lower spring constant is the cause of a slower response time into a separated state between the electrodes. This has been a critical problem in the past reported RF-MEMS switches. A pull-up electrode approach above the membrane is one of solutions to this problem, but a complicated structure increases the number of processes and process cost. Vertical comb-drive approach is reported to achieve a fast pull-up response using a simple structure with two metal layers [14,15]. Lateral comb-drive actuators integrated in circuit are also reported, and actuation voltage is still over 10 V [16,17].

This paper presents that the laterally movable triple electrodes actuator embodied with a simple and low-cost one metal layer process is effective for a low voltage actuation and fast response in RF-MEMS switches.

## 2. Design

### 2.1. Effect of Laterally Movable Triple Electrodes Actuator

**Figure 1:**
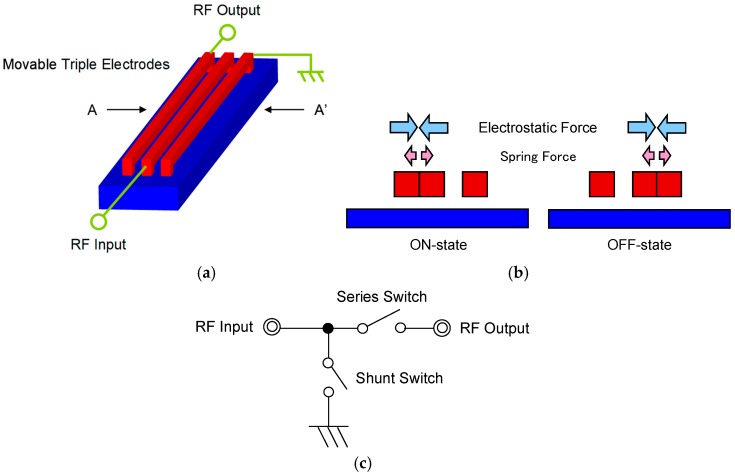
Schematic diagram of the radio frequency micro-electro-mechanical systems (RF-MEMS) switch using laterally movable triple electrodes actuator: (**a**) overview; (**b**) cross-sectional view along A–A’; and (**c**) equivalent circuit.

The schematic diagram of the switch as described in this paper is shown in Figure 1a overview and Figure 1b cross-sectional view. In the on-state, actuation voltage is applied between the center electrode and left electrode and these two electrodes come into contact. The RF signal goes through from the input port to the output port. In the off-state, the center electrode and right electrode come into contact and the RF signal goes through to the ground. The RF signal is shut, not to be transmitted to RF output port. An equivalent circuit of the switch is shown in Figure 1c. The series and shunt type switches can be expressed on one switch. The series switch consists of the center electrode and the left electrode connected to RF input port and RF output port, respectively. On the other hand, the shunt switch consists of the center electrode and the right electrode connected to RF input port and the ground. The series switch is closed, and the shunt switch is opened in the on-state. To reverse in the off-state, the connection to the ground enables high isolation in spite of a small gap between the electrodes on the series switch. The small gap makes the response time shorter at a low actuation voltage, because the electrostatic force depends inversely on the gap by the factor of 2.

The triple electrodes are allowed to move laterally by electrostatic forces in not only the on-state but also the off-state, thus the restoring spring force can be reduced. A spring constant of the electrodes small enough to reduce pull-in voltage must be chosen. According to an expression for fixed-fixed beam, the spring constant *k* of the electrodes depends on physical dimensions, residual stress, and intrinsic material stiffness, given by the following [18]:

(1) $$k=32Ew{\left(\frac{t}{l}\right)}^{3}+8\sigma \left(1-\nu \right)w\left(\frac{t}{l}\right),$$

where *w*, *t*, and *l* are width, thickness, and length, respectively; *E* is the Young’s modulus; *v* is the Poisson’s ratio; and *σ* is the residual stress of the movable electrodes. The length is effective in order to reduce the spring constant because the spring constant depends inversely on the length by the factor of 3 in the case of low residual stress. The residual stress is determined to be a practical value of material under *σ* = 100 MPa to achieve actuation a voltage of 7 V and a response time of 5 µs. The residual stress was evaluated from the mechanical resonant frequency of the movable electrodes, measured by laser Doppler interferometer.

**Figure 2:**
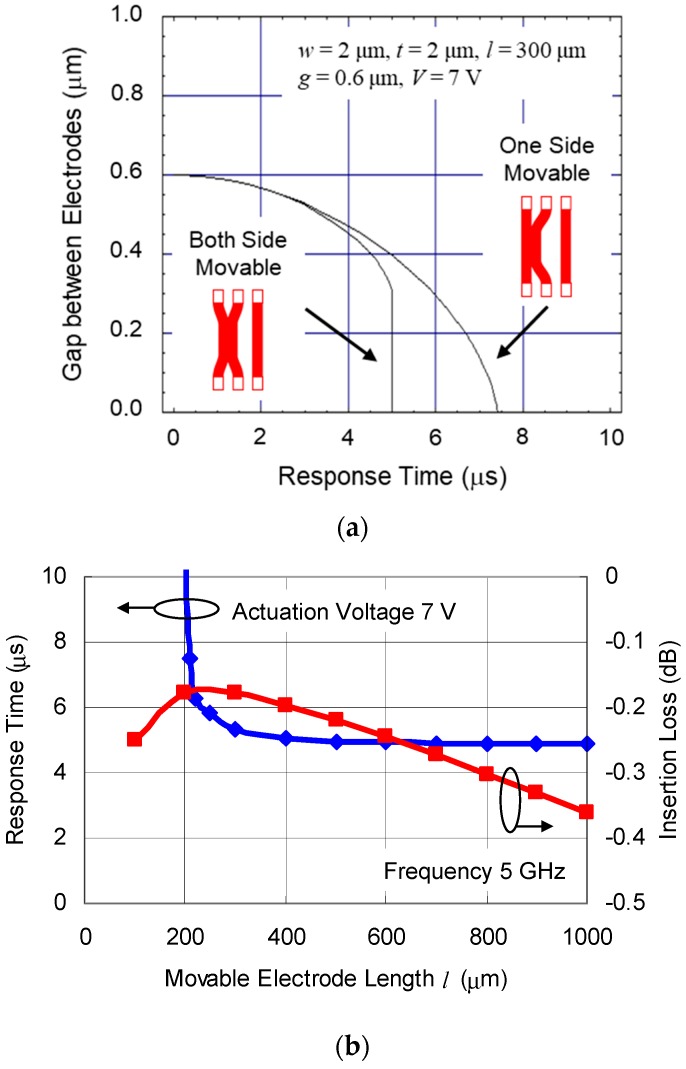
(**a**) Comparison of simulated response time between case of both side of electrodes movable and one side movable; (**b**) simulated response time and insertion loss as a function of length of movable electrode.

A comparison of simulated response time between the case of both sides of the electrodes movable and one side movable is shown in Figure 2a. The actuation characteristics are calculated with dynamic analysis equation described in the work of [19]. The calculation parameters are *w* = 2 µm, *t* = 2 µm, *l* = 300 µm, gap between electrodes *g* = 0.6 µm, and actuation voltage *V* = 7 V. The response time is 5 µs in the case of both electrodes movable, which is nearly 1.5 times shorter than 7.4 µs in the case of one side movable. The travel distance of each electrode becomes half of the case of one side movable, this configuration is effective for a low voltage actuation and fast response.

### 2.2. Movable Electrode as RF Transmission Line

Not only the actuation characteristics, but also the RF characteristic is essential to optimize the movable electrode dimension. The simulated response time and insertion loss as a function of length of the movable electrode are shown in Figure 2b. An electromagnetic field analysis (Agilent Momentum) is used for the insertion loss simulation. The calculation parameters are *w* = 2 µm, *t* = 2 µm, *g* = 0.6 µm, and the dielectric material inserted between two signal lines is Al_2_O_3_ (thickness *t*_d_ = 10 nm, dielectric constant *ε*_r_ = 10). The insertion loss of the series switch depends on capacitance determined by lateral overlap area between the movable electrodes. If the length becomes longer, the insertion loss decreases as a result of the increase of the capacitance. The response time becomes shorter as a result of the decrease of the spring constant, according to Equation (1). Beyond a certain length, as the membrane gets longer, the insertion loss increases again as a result of the increased resistance of the electrodes. In this situation, the capacitive coupling loss is not more than the resistive loss. The geometry needs to satisfy both the actuation characteristics and the RF characteristics in order to optimize the movable electrode dimensions. The length of the movable membrane *l* was optimized to 500 µm, at the same time minimizing the insertion loss from the transmission lines −0.22 dB at 5 GHz and the response time 5 µs at the actuation voltage of 7 V.

**Figure 3:**
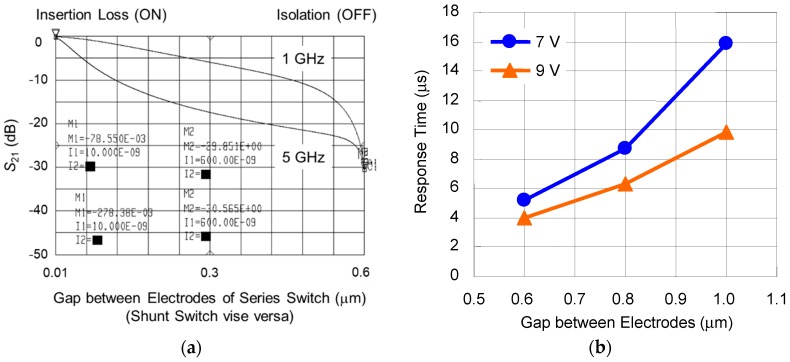
(**a**) Simulated RF characteristics as a function of gap between electrodes of series shunt hybrid switches; (**b**) simulated response time as a function of gap between electrodes.

The simulated RF characteristics as a function of gap between electrodes of series shunt hybrid switches is shown in Figure 3a, which is done using a high frequency circuit simulator (Agilent ADS). The insertion loss is lower than −0.5 dB and the isolation is higher than −30 dB, including the loss of the transmission lines from 1 to 5 GHz. The simulated response time as a function of gap between electrodes is shown in Figure 3b. The response time is shortened to 4 µs at the higher actuation voltage 9V in the case of the designed gap of 0.6 µm.

### 2.3. Summary of Design

The results of design predict that the movable triple electrodes actuator has a potential toward low voltage actuation and fast response in RF-MEMS switches. The designed specifications are summarized in Table 1.

## 3. Fabrication

### 3.1. Process Flow

**Figure 4:**
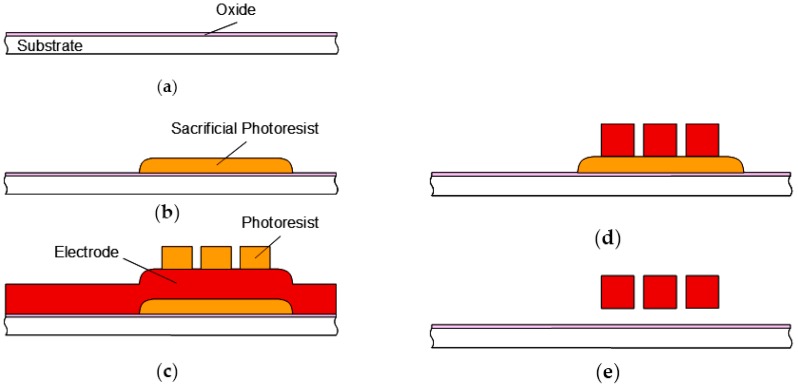
Fabrication process flow for sputtered-Al switch, (**a**) formation of isolation oxide on substrate; (**b**) formation of sacrificial photoresist; (**c**) Al deposition for electrodes, and photoresist formation; (**d**) Al etching; (**e**) release of electrodes as actuator.

The fabrication process flow for the switches is shown in Figure 4. The structures were fabricated using a simple, low-cost two-mask process. The switches were fabricated on the high-resistivity silicon substrate (2000–20,000 Ω⋅cm) with an isolation oxide layer. A photoresist was spin-coated and patterned to create a sacrificial layer. Post-bake with hotplate and DUV (deep ultraviolet) were implemented to smooth the edges of the photoresist. The movable triple electrodes were fabricated by sputtering 2 µm aluminum alloy (Al–1%Si–0.5%Cu) and patterned. The 0.6 µm air gaps between the electrodes were patterned with photolithography using i-line stepper and etched using RIE (reactive ion etching). Thick photoresist was used in order to etch the thick aluminum layer. Finally, the sacrificial photoresist was removed using oxygen plasma to eliminate stiction of the beams. The aluminum electrodes are covered with Al_2_O_3_ native oxide dielectric layer.

**Figure 5:**
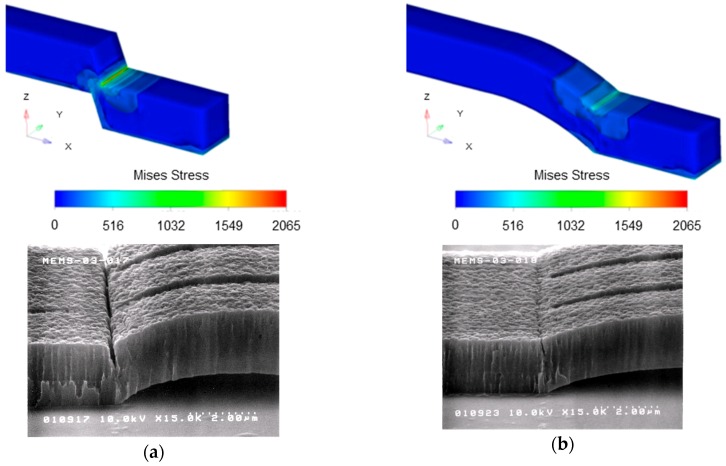
Simulated stress and scanning electron microscope (SEM) images of support area at post-bake temperature for sacrificial photoresist: (**a**) 150 °C; (**b**) 180 °C.

The stress simulation results using FEM (finite element method, CoventorWare) are shown in Figure 5. The stress is concentrated to the support area because of the tensile stress of the fixed-fixed beams. It is revealed that the stress concentration can be decreased in case of smaller angle between the ground and the support using smooth edges of the photoresist formed with higher temperature post-bake. Structural breakdown was observed at the stress concentrated part in SEM (scanning electron microscope) images in Figure 5a. The angle between the ground and the support 45° by 150 °C post-bake was not sufficient, the temperature of the post-bake was adjusted in order to make the optimal shape of the sacrificial photoresist, 30° by 180 °C post-bake. In order to avoid the stress concentration, wider supports were also effective. The triple electrodes structures were fabricated by the stress control in the beams and the supports.

### 3.2. Structure Observation

**Figure 6:**
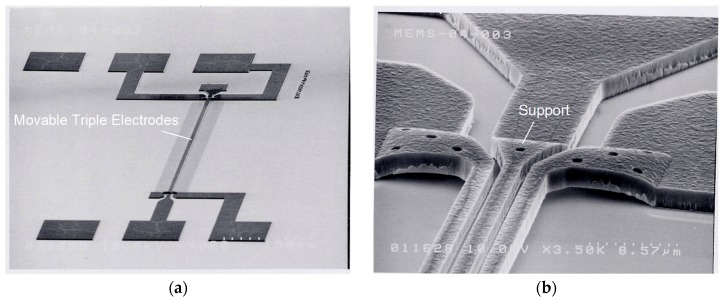
SEM images of fabricated switches: (**a**) overview; (**b**) close-up of support area.

SEM images of the fabricated switches are shown in Figure 6a overview and Figure 6b close-up of the support area. The laterally movable triple electrodes actuator for RF-MEMS switches reflected the geometry specifications shown in Table 1.

## 4. Measurement Result

**Figure 7:**
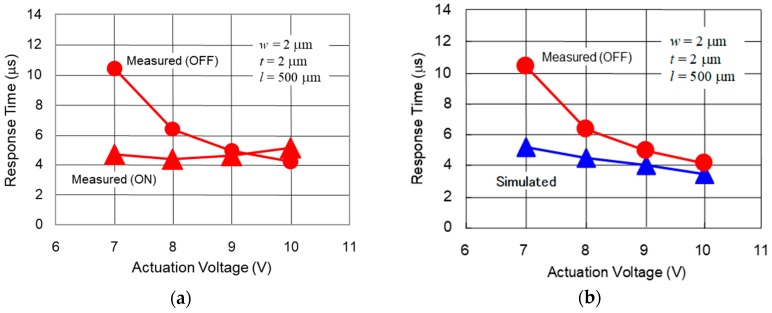
(**a**) Measured response time as a function of actuation voltage; (**b**) comparison with simulation in the off-state.

The response time of the fabricated switches was measured using the control voltage applied to the center electrode with 1 kHz rectangular wave form. The applied control signals are summarized in Table 2. The high control signal means the actuation voltage, while the low one means the grounded 0 V. The bistable actuation consumes more power than monostable, but less than thermoelectric actuators [10,13]. The measured response time as a function of the actuation voltage is shown in Figure 7a. The response time and the actuation voltage were 4.9 µs at 7.0 V in the on-state, which was in good agreement with the simulation. The characteristics in the off-state was 10.5 µs at 7.0 V. A comparison with the simulation is shown in Figure 7b. A higher voltage than 9.0 V is necessary to achieve a response time of 5.0 µs. 

**Figure 8:**
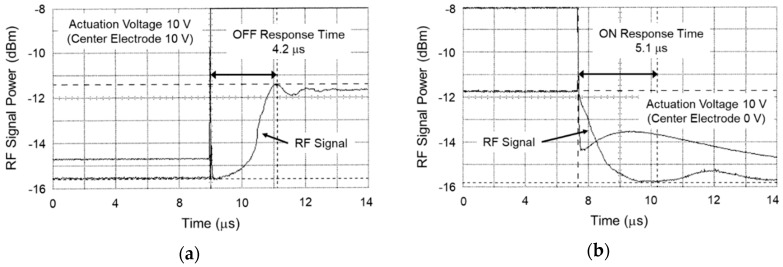
Measured RF signal as a function of time on shunt switch: (**a**) on-state; (**b**) off-state.

The reason for the deviation between the measured and the simulated actuation voltage in the off-state is considered to be larger gap between the center and the right electrodes. The measured gap using laser microscopy was around 0.8 µm, which was 0.2 µm lager than the geometry specifications caused by deformation of the electrodes as a result of stress distribution in asymmetric support structure. The actuation voltage of 9.0 V was necessary to achieve a response time of 5.0 µs in the case of the gap between the electrodes of around 0.8 µm, which was in agreement with the simulated results in Figure 3b. The measured RF signal as a function of time is shown in Figure 8. It was confirmed that the response time became shorter 4.2 µs at actuation voltage 10.0 V on the shunt switch in the off-state.

## 5. Conclusions

This paper presents the design, fabrication, and testing of laterally movable triple electrodes actuator in capacitive series shunt hybrid RF-MEMS switches for a low voltage actuation and fast response. The actuator allows all of the suspended beams to move laterally in two directions entirely by electrostatic forces. The structure was optimized considering the RF and mechanical characteristics. The switches were fabricated using a simple, low-cost two-mask process with sputtered aluminum as the structural material. The measured response time and the actuation voltage were 5.0 µs and 9.0 V, respectively. The experimental results were in good agreement with the simulated actuation characteristics. Consequently, it was revealed that the laterally movable triple electrodes actuator is effective toward the lower actuation voltage than 10 V and the faster response than 10 µs in RF-MEMS switches. The advantage is summarized in comparison with reported works in Table 3.

For further work, improvement of roughness on the sidewall of the electrodes should be done for good electrical contact and RF characteristics. Mechanical fatigue should be also taken into consideration. A reliability test will be done to confirm that the bistable actuation is effective to avoid stiction of the electrodes.

It is expected that the laterally movable triple electrodes actuator advances RF-MEMS switches, switched capacitors, and MEMS devices in circuits for wireless sensor networks.

## Figures and Tables

**Table 1:** Designed Specifications of Movable Triple Electrodes Actuator in radio frequency micro-electro-mechanical systems (RF-MEMS) switches.

**Movable Electrodes**		
Length (*l*)		500 µm
Width (*w*)		2 µm
Thickness (*t*)		2 µm
Gap (*g*)		0.6 µm
Actuation Voltage		7 V
Response Time		5 µs
**RF Frequency**	**Insertion Loss**	**Isolation**
1 GHz	−0.3 dB	−30.1 dB
5 GHz	−0.5 dB	−30.8 dB
Gap (*g*) Series Switch (Shunt vise versa)	0.01 µm	0.6 µm

**Table 2:** Applied control signals of movable triple electrodes actuator.

		Left Electrode	Center Electrode	Right Electrode
	
**ON**		High	Low	Low
**OFF**		High	High	Low

**Table 3:** Comparison of low voltage actuators in RF-MEMS switches.

	Ref. [8]	Ref. [9]	Ref. [10]	Ref. [11]	Ref. [12]	This Work
Actuator Type	Vertical Monostable ON: ES OFF: MR Au	Vertical Monostable ON: ES OFF: MR Au	Lateral Monostable ON: ET OFF: MR Poly-Si	Lateral Monostable ON: ES OFF: MR Si	Lateral Bistable ON: ES OFF: ES Si	Lateral Bistable ON: ES OFF: ES Al
Switch Type	Ohmic series	Capacitive shunt	Ohmic series	Ohmic series	Ohmic series	Capacitive series shunt hybrid
Actuation Voltage	0.5 V	4.8–6.2 V	2.5–3.5 V	15 V	57 V	9.0 V
Response Time	500 µs	33–37 µs	300 µs	120 / 500 µs	56 / 40 µs	5.0 µs
Footprint Size	1264 × 635 µm^2^ (Incl. pads)	300 × 180 µm^2^	200 × 100 µm^2^	2.55 × 2.39 mm^2^	1.4 × 1.4 mm^2^	550 × 50 µm^2^
RF Frequency	3k–3 GHz	20–40 GHz	40–50 GHz	DC–10 GHz	DC–10 GHz	1–5 GHz

ES: Electrostatic, ET: Electrothermal, MR: Mechanical Restoring.
